# YKL-40 Protein Correlates with the Phenotype of Asthma

**DOI:** 10.1007/s00408-015-9693-y

**Published:** 2015-02-08

**Authors:** Krzysztof Specjalski, Marta Chełmińska, Ewa Jassem

**Affiliations:** Department of Allergology, Medical University of Gdansk, Ul. Debinki 7, 80-952 Gdansk, Poland

**Keywords:** Asthma phenotypes, Asthma biomarkers, YKL-40, Chitinase-like protein

## Abstract

**Purpose:**

YKL-40 is a chitinase-like protein found to correlate with asthma as well as numerous infectious and autoimmune diseases or cancer. The aim of the present study was to investigate the role of YKL-40 as a possible marker of asthma and its associations with factors differentiating phenotypes of asthma.

**Methods:**

The study group comprised 167 patients, including 116 women and 51 men aged 18–88 years with chronic asthma. The control group comprised 81 healthy individuals, including 50 women and 31 men aged 19–86 years. In every participant, medical history was taken; spirometry and skin prick tests were performed. YKL-40 was determined in sera by means of ELISA test.

**Results:**

Mean serum YKL-40 level was 59.7 ng/ml (53.6–65.7 ng/ml; 95 % CI) with significant difference between asthmatics and healthy controls (mean values: 66.8 ± 53.8 vs. 44.9 ± 29.4 ng/ml; *p* < 0.001). In asthmatics, the level was significantly higher in subgroup with poor control of symptoms and exacerbations (91.8 ± 57.1 ng/ml) compared to stable asthmatics (59.6 ± 50.8 ng/ml; *p* < 0.001) as well as in atopic compared to non-atopic asthmatics (77.2 ± 53.9 vs. 61.1 ± 57.8 ng/ml; *p* < 0.001). Mean YKL-40 level in obese asthmatics was 135.6 ng/ml compared to 50.0 ng/ml in non-obese (*p* < 0.001). When phenotypes of early-onset atopic, late-onset non-atopic, and obesity-related asthma were compared, YKL-40 levels were 80.62 ± 46.9, 51.5 ± 24.9, and 168.1 ± 71.5 ng/ml, respectively (*p* < 0.05).

**Conclusion:**

Although YKL-40 is not a specific marker for asthma, it correlates with some clinical features such as exacerbation, level of control, atopy, and obesity.

## Introduction

Asthma is defined as a chronic disorder of airways with inflammation and bronchial hyperresponsiveness as major underlying phenomena [1]. For many years, asthmatics have been regarded as a heterogeneous population, and as a consequence, asthma has been a vague term describing a group of several clinical symptoms rather than a single pathologic process. Some decades ago, this was reflected in the concept of extrinsic and intrinsic asthma. The former was usually developed in childhood, was atopic with identifiable allergens affecting course of the disease, and was accompanied by other atopic diseases. The latter was developed later in the lifetime, more often by women, and was not associated with allergy [2]. As this division did not make it possible to predict the response to treatment or the course of the disease, it was abandoned [3]. However, in recent years, the concept of subtypes of asthma has been debated once again with proposals of phenotypes (observable properties of an organism produced by interactions of the genotype and the environment) and endotypes (pathological pathways explaining properties of phenotype) [4, 5]. Although there is no generally accepted consensus on phenotypes of asthma, some variables differentiate asthmatics strongly in many studies, including age of asthma onset, atopy, gender, obesity, sputum eosinophilia or neutrophilia, etc. On their basis, the following phenotypes have been proposed: early-onset allergic, late-onset eosinophilic, exercise-induced, obesity-related, neutrophilic [6]. Better understanding of asthma phenotypes and endotypes seems crucial at the dawn of the era of biologicals. As there is no general pattern of asthmatic inflammation, efficacy of given drug may vary between phenotypes. Potential effect of therapy should be monitored with the use of biomarkers specific for the given phenotype.

Early-onset allergic asthma is a phenotype that has been described the best. It is associated with Th2-type inflammation and is often accompanied by other atopic diseases, such as allergic rhinitis or atopic dermatitis. Sputum eosinophils and FeNO are considered good biomarkers of allergic asthma as they correlate with clinical outcome and therapy with inhaled steroids [7, 8]. Cytokines involved in Th2-type inflammation are IL-4, 5, and 13 [9]. In contrast to early-onset allergic asthma, phenotypes not related to Th2-type inflammation have not been precisely defined e.g., there are no generally accepted criteria for diagnosing obesity- or exercise-induced asthma. Little is also known about their immunological and inflammatory underpinnings. No biomarkers have been introduced so far into everyday practice.

In recent years, several questions have been raised on the role of chitinases and chitinase-like proteins (CLPs) in chronic bronchial inflammation. Among the CLPs, the role of YKL-40 has been intensively investigated. YKL-40 was found to correlate with asthma but is not specific with high serum levels in numerous infectious diseases and malignancies [10–13]. In studies on asthma, some authors found dubious relations with clinical features and biomarkers (FEV_1_, severity level, eosinophilia, etc) [10, 14, 15]. It is not known whether YKL-40 is typical for some phenotypes of asthma. However, this hypothesis may be confirmed by studies showing its high concentration in allergic inflammation [16, 17].

The aim of the present study was to investigate the role of YKL-40 as a possible marker of asthma and its associations with factors differentiating phenotypes of asthma.

## Materials and Methods

The study group comprised 167 patients, including 116 women and 51 men aged 18–88 years (mean age: 50.1 years) with chronic asthma, confirmed by reversibility test and treated for at least 12 months. Patients were recruited in the In- and Out-patient Departments of Allergology, Medical University of Gdansk, Poland, from March 2013 to February 2014. Exclusion criteria were episodic asthma, concomitant chronic respiratory disease (COPD, interstitial disease, etc.), cancer diagnosed or treated within previous 5 years, current immunosuppression or immunodeficiency, and hepatic or renal insufficiency. Pregnant women also were not eligible for the study.

The control group comprised 81 healthy individuals, including 50 women and 31 men aged 19–86 years (mean age: 48.6 years). They were recruited among patients and employees of the University Clinical Centre, Gdansk, Poland. Exclusion criteria included: chronic respiratory disorders, current or past history of allergy, and all chronic diseases mentioned in reference to study group.

Characteristics of study groups have been shown in Table 1.

**Table 1:** Characteristics of the study groups

	Asthma group	Control group	*p* value
Age (years)	50.1 ± 15.3	48.6 ± 15.3	0.44
Gender (women:men)	116:51	50:31	0.22
FEV_1_/FEV_1_ predicted (%)	79.9 ± 20.0	99.44 ± 9.7	**<0.001**
BMI (kg/m^2^)	25.7 ± 3.1	25.1 ± 3.2	0.64
White blood count (10^9^/l)	8.1 ± 3.5	6.67 ± 2.2	**<0.001**
Number of eosinophils (10^9^/l)	0.37 ± 0.85	0.16 ± 0.16	**0.03**
Number of neutrophils (10^9^/l)	5.61 ± 7.3	5.07 ± 10.0	0.63
CRP (mg/l)	4.7 ± 9.8	2.1 ± 3.8	**0.02**
Positive skin prick tests	83	0	N/A

Data are presented as mean values ± SD

Bold values are statistically significant (*p* < 0.005)

### Study Protocol

Having given informed consent, all the subjects underwent clinical assessment, skin prick tests, and spirometry (*Lungtest 1000*, MES, Poland). Medical history was analyzed for information on asthma onset, atopy, coexisting diseases, etc. Level of asthma control was assessed according to GINA 2012 guidelines [1]. Exacerbation of asthma was defined as worsening of patients’ condition requiring hospitalization or introduction of systemic steroids (in patients taking systemic steroids regularly—increase of the dose). As the precise date of asthma diagnosis was impossible to acquire in the vast majority of cases, onset of the disease was recorded in one of four options: 12 years of age or earlier (early onset), 13–30, 31–40 years, and after 40th year of life (late onset). Diagnosis of Churg–Strauss syndrome was based on ACR criteria.

In order to determine atopic status, skin prick tests were performed with common airborne allergens: grass, trees, weeds, cat, dog, *Dermatophagoides farinae*, *Dermatophagoides pteronyssinus*, and molds (*Allergopharma,* Reinbek, Germany). Atopy was defined as at least one positive result (mean wheal diameter ≥3 mm).

Consecutively, three samples (15 ml) of venous blood were collected from every participant. 5 ml of blood was centrifuged and serum was stored at the temperature of −20 °C for 1–7 months. YKL-40 was detected by means of ELISA test (*Quidel*, San Diego, USA.) in accordance with manufacturer’s instructions. The remaining samples were used for determination of CRP and total blood count.

All data were analyzed using Statistica 10.0 statistical software (*Stat Soft*, Tulsa, USA). Demographic data were analyzed with *χ* ^2^ test or *t* test. Concentration of YKL-40 was compared in asthmatics and controls as well as in subgroups of asthmatics. For these analyses, U Mann–Whitney’s test was applied. Correlations between YKL-40 and laboratory data were assessed with Pearson’s test. Level of significance was defined as *p* ≤ 0.05.

Study protocol was approved by the Local Bioethics Committee at the Medical University of Gdańsk (no. NKBBN 396/2013) and was performed in accordance with the ethical standards of the Declaration of Helsinki.

## Results

Out of 167 patients, symptoms of asthma were found to be controlled in 49 (29.3 %), partly controlled in 37 (22.1 %), and uncontrolled in 81 (48.6 %) patients. Patients’ condition was assessed as stable in 130 cases, and criteria of exacerbation were met in 37 cases. Atopy was found in 83 patients with sensitizations to house dust mites, grass, and weeds as the most common. In 12 cases, there was aspirin hypersensitivity confirmed by oral provocation test. In nine patients, Churg–Strauss syndrome was diagnosed either before or shortly after the blood collection.

Reported time of asthma onset was ≤12 years of age—42 patients; 13–30 years—40; 31–40 years—33; and >40 years—52.

Mean serum YKL-40 level was 59.7 ± 48.2 ng/ml (53.6–65.7 ng/ml; 95 % CI) with significant difference between asthmatics and healthy controls (mean values 66.8 ± 53.8 vs. 44.9 ± 29.4 ng/ml; *p* < 0.001). In the whole group of study participants, there was weak correlation with age (*r* = 0.24; *p* < 0.05) or gender (women vs. men: mean 61.3 ± 50.5 vs. 56.3 ± 43.6 ng/ml, respectively; *p* = 0.14) and moderate correlation with BMI (*r* = 0.44; *p* < 0.05).

**Fig. 1:**
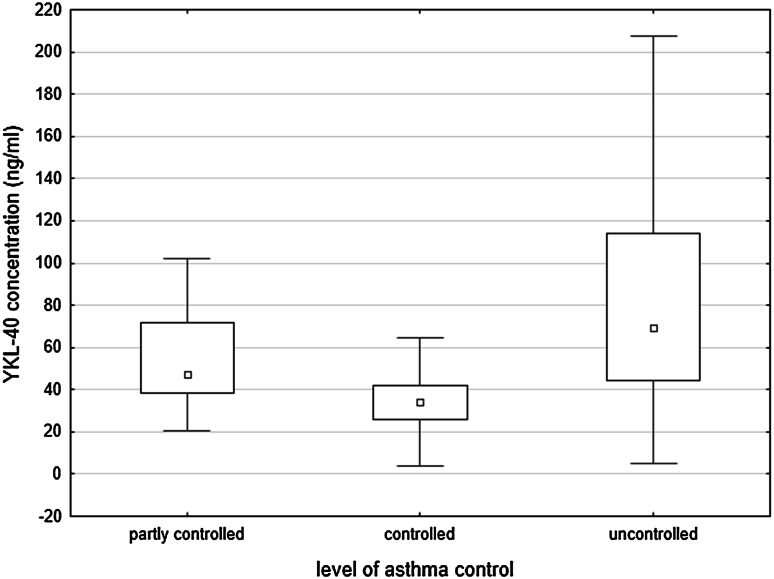
YKL-40 levels in relation to level of asthma control. Data are presented as median (*central square*), 25th and 75th percentiles (*top* and *bottom* of *boxes*), 10th and 90th percentiles (*top* and *bottom* of *bars*)

In asthmatics, serum concentration of YKL-40 was significantly higher in subgroup with poor control of symptoms (Fig. 1). YKL-40 levels were also higher in patients with asthma exacerbation compared to patients with the stable disease (91.8 ± 57.1 vs. 59.6 ± 50.8 ng/ml; *p* < 0.001). The full comparison of exacerbation and stable groups is presented in Table 2.

**Table 2:** Comparison of patients with stable asthma and exacerbation

	Stable asthma	Asthma exacerbation	*p* value
Age (years)	49.7 ± 14.7	51.8 ± 17.4	0.49
Gender (women:men)	89:41	27:10	0.59
FEV_1_/FEV_1_ predicted (%)	83.7 ± 18.4	66.5 ± 19.8	**<0.001**
White blood count (10^9^/l)	7.5 ± 2.4	10.1 ± 5.3	**<0.001**
Number of eosinophils (10^9^/l)	0.41 ± 0.9	0.22 ± 0.29	0.07
Number of neutrophils (10^9^/l)	5.2 ± 7.8	7.1 ± 5.4	**<0.001**
CRP (mg/l)	4.2 ± 10.4	6.4 ± 7.0	**<0.001**
YKL-40 (U/l)	59.6 ± 50.8	91.8 ± 57.1	**<0.001**

Data are presented as mean values ± SD

Bold values are statistically significant (*p* < 0.005)

**Fig. 2:**
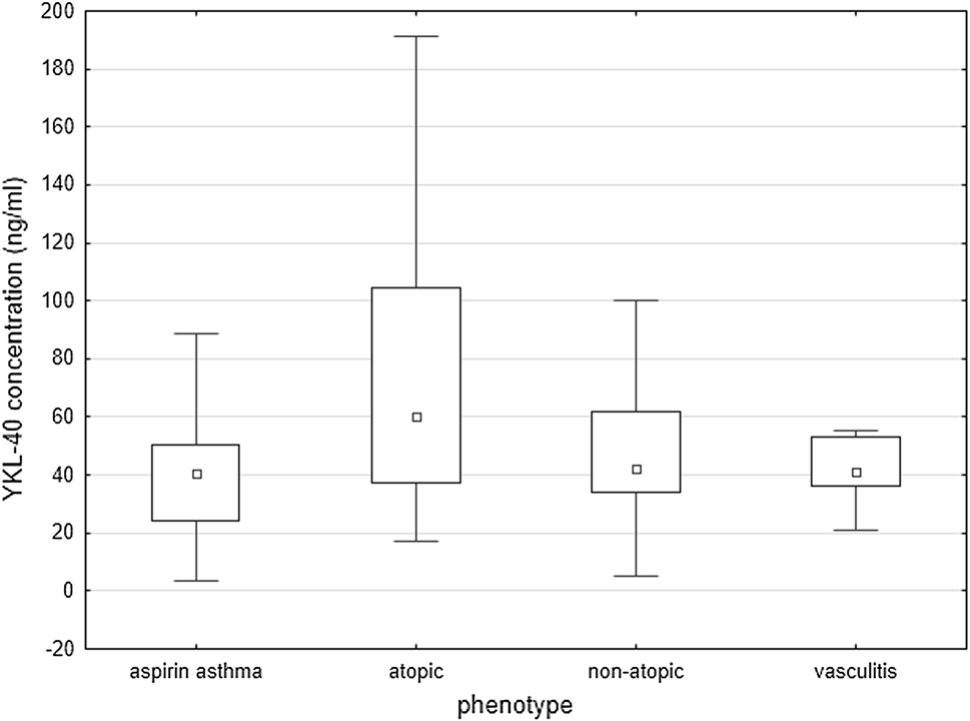
YKL-40 levels in relation to asthma phenotypes: aspirin asthma, atopic asthma, non-atopic asthma, asthma accompanying vasculitis. Data are presented as median (*central square*), 25th and 75th percentiles (*top* and *bottom* of *boxes*), 10th and 90th percentiles (*top* and *bottom* of *bars*)

**Fig. 3:**
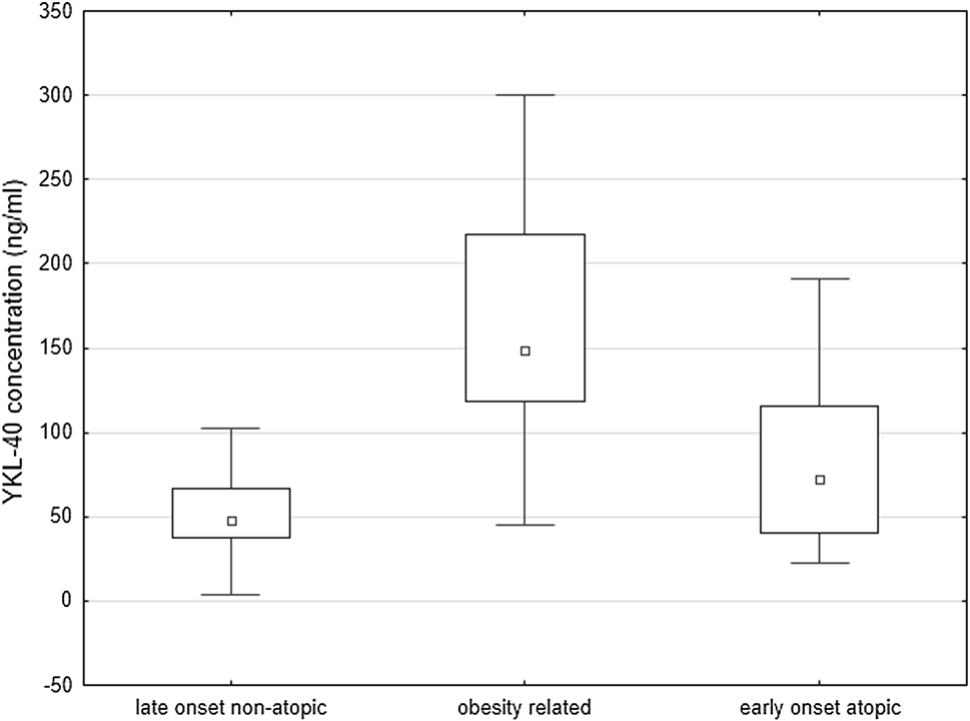
YKL-40 levels in relation to phenotypes of asthma: late-onset non-atopic, obesity-related, and early-onset atopic. Data are presented as median (*central square*), 25th and 75th percentiles (*top* and *bottom* of *boxes*), 10th and 90th percentiles (*top* and *bottom* of *bars*)

Significant differences were found in YKL-40 concentration in relation to the phenotype of asthma. When the division into atopic, non-atopic, aspirin, and vasculitis-associated asthma was applied, the highest levels were found in atopic subgroup (Fig. 2). Slightly different findings were revealed when the analysis was limited to three subgroups with strictly defined phenotypes described previously: early-onset atopic, late-onset non-atopic, and obesity-related [6]. Although the difference between early-developed atopic and late-onset non-atopic asthma was still significant, the highest values were observed in cases of obesity-related asthma (80.62 ± 46.9, 51.6 ± 24.9 vs. 168.1 ± 71.5 ng/ml, respectively; Fig. 3). In fact, in the whole asthmatic group, obesity was an important determinant of YKL-40 level, as its mean value in obese asthmatics was 135.6 ng/ml compared to 50.0 ng/ml in non-obese (*p* < 0.001). In non-obese asthmatics, YKL-40 level was similar to controls (50.0 vs. 44.9 ng/ml; *p* = 0.14). Difference between obese and non-obese non-asthmatics was larger (54.2 vs. 43.9 ng/ml) but still not significant (*p* = 0.07). Conversely, no correlations with blood eosinophilia, neutrophilia, or CRP were found.

## Discussion

Chitinases are evolutionarily conserved hydrolases. By degrading chitin, they play an important role in controlling homeostasis in lower life forms—crabs, insects, and spiders. In contrast to chitinases, CLPs bind chitin but do not have degrading activity. Mammalian chitinases and CLPs (acidic mammalian chitinase, chitotriosidase, oviductin, and human cartilage glycoprotein-HcGP/YKL-40) are produced by monocytes, macrophages, and neutrophils in response to parasitic or fungal infection [18]. Elevated levels of YKL-40 were also observed in numerous pathologies, including rheumatoid arthritis, myocardial infarction, diabetes, and several types of cancer [11, 13, 19]. Their role in the pathogenesis of these diseases is still not understood. However, expression at inflammation sites suggests that they play a role in antiparasitic defense and repair mechanisms.

Difference in YKL-40 levels between asthmatics and healthy controls has been demonstrated in several studies [10, 14]. However, the increase is not specific, and there are studies showing no correlations in some groups of asthmatics [20]. As asthma is a heterogenous disease, it may lead to the hypothesis that YKL-40 is elevated only in some of its phenotypes.

In this study, we confirmed increased level of YKL-40 in asthmatics and its correlation with clinical course of the disease. The higher levels were found in patients with current exacerbation and long-term poor control of symptoms. This is in line with previous studies showing correlations with several clinical characteristics. Konradsen found the highest concentrations of YKL-40 in patients with severe therapy-resistant asthma with elevated FeNO [21]. Dura revealed an increase during exacerbation and reverse correlation with FEV_1_ [22]. Tang found correlation with exacerbation, FEV_1_, and blood eosinophilia [14].

Significant differences were revealed when patients with asthma were divided into subgroups varied in terms of disease onset, atopy, aspirin hypersensitivity, obesity etc. Owing to the lack of one, generally accepted classification of phenotypes and endotypes of asthma, we initially divided patients into four groups: atopic, non-atopic, aspirin asthma, and asthma coexisting with vasculitis. We found that atopic asthma was characterized by the highest serum levels of YKL-40. Consecutively, we applied modified classification proposed by Wenzel to select “the most typical” patients with early-onset (<12 years of age) atopic, late-onset (>40 years of age) non-atopic, and obesity-associated asthma [6]. Only three phenotypes were compared as we did not analyze sputum, and as a consequence, differentiating neutrophilic and eosinophilic pattern of inflammation was not possible. In this comparison, the highest mean value was found in obesity-related asthma followed by atopic asthma.

Relations between chitinases and atopy have already been investigated several times. That started with the observation that reaction to parasitic infection is in some aspects similar to mechanisms of allergic inflammation, which would explain the role of chitinases in both processes. YKL-40 has been found to facilitate allergen sensitization and IgE production. Its concentration was elevated in bronchoalveolar lavage fluid after allergen challenge [23]. In the study on the murine asthma model, acidic mammalian chitinase (AMC) was found to be upregulated and its expression was induced by cytokines IL-4 and IL-13. Inhibition of AMC activity prevented from airway hyperresponsiveness [24]. In another animal model, oral administration of chitin downregulated Th2 inflammation [25].

Bronchial biopsies provided data on associations between YKL-40 and structural changes in the tissues. YKL-40 was found to promote proliferation of bronchial smooth muscle cells, which is one of the characteristics of bronchial remodeling [26]. Its serum and bronchial lavage fluid level correlates with subepithelial membrane thickness [27]. Some significant correlations have also been revealed in genetics of YKL-40. This protein is encoded by the chitinase-3-like 1 gene (CHI3L1). Single nucleotide polymorphisms in CHI3L1 promoter were found to be associated with elevated YKL-40 levels as well as atopy, asthma, and bronchial hyperresponsiveness [28, 29].

On the other hand, it needs to be emphasized that in many papers, YKL-40 was also found elevated in patients with COPD, who have pattern of inflammation not related to Th2 mechanisms, and smokers who had not developed this disease [12]. There were some studies showing that circulating YKL-40 was associated with levels and decline in lung function in the general population. Their authors hypothesized that this could be a biomarker of susceptibility to the long-term effect of cigarette smoking [30].

Obesity was found to correlate with YKL-40, and in the group of obesity-related asthma, we found the highest mean level. It is generally recognized that obesity has substantial impact on asthma. However, whether it is a driving factor initiating the disease, a mere confounder or just a frequent co-morbidity is debatable. Surely, obesity may enhance the perception of breathlessness in asthmatics due to greater energy expenditure while breathing. By leading to gastroesophageal reflux, it is associated with chronic cough. In contrast, in severe asthma requiring systemic steroids, obesity is one of their common side-effects rather than the cause of the pathology. Many authors emphasize associations between obesity and general inflammation with elevated levels of TNFα, IL-6, and leptins [31, 32]. Pro-inflammatory tendency in obese asthmatics may be another explanation for high levels of YKL-40.

## Conclusion

Asthma is a heterogeneous disease with several phenotypes reflecting varied inflammatory patterns. It seems that chitinases are upregulated mostly in atopic asthma and in obese patients. However, the level of YKL-40 correlates with clinical features (exacerbation, lack of control, and FEV_1_) in the whole asthmatic population.

Although not specific for asthma, YKL-40 could have its practical application in assessment of disease control and phenotype. Further studies would be useful to assess whether monitoring of its levels could facilitate making successful clinical decisions.
